# CD73 Promotes Chronic Lymphocytic Leukemia

**DOI:** 10.3390/cancers14133130

**Published:** 2022-06-26

**Authors:** David Allard, Pavel Chrobak, Yacine Bareche, Bertrand Allard, Priscilla Tessier, Marjorie A. Bergeron, Nathalie A. Johnson, John Stagg

**Affiliations:** 1 Centre de Recherche du Centre Hospitalier de l’Université de Montréal, Montréal, QC H2X 0A9, Canada; david.allard.1@umontreal.ca (D.A.); pavel.chrobak@hotmail.com (P.C.); yacine.bareche@umontreal.ca (Y.B.); bertrandallard@gmail.com (B.A.); priscilla_tessier@hotmail.com (P.T.); marjorie-allison.bergeron.1@ulaval.ca (M.A.B.); 2 Institut du Cancer de Montréal, Montréal, QC H2X 0A9, Canada; 3 Faculté de Pharmacie, l’Université de Montréal, Montréal, QC H3T 1J4, Canada; 4 Department of Medicine, Jewish General Hospital, Montréal, QC H3T 1E2, Canada; nathalie.johnson@mcgill.ca

**Keywords:** CD73, adenosine, chronic lymphocytic leukemia, PD-L1

## Abstract

**Simple Summary:**

Many patients with chronic lymphocytic leukemia (CLL) still fail current therapies. CD73 is a novel therapeutic target for solid tumors, but its role in CLL remains unclear. The aim of our study was to investigate the therapeutic potential of targeting CD73 in CLL. Using genetically engineered mice, our study reports a pro-leukemic role for CD73 in an autochthonous mouse model of CLL. Furthermore, we observed an association between PD-L1 expression on CLL cells and adenosine signaling according to sex. Our findings provide a rationale for targeting CD73 in CLL in combination with anti-PD-1/PD-L1 immunotherapies and suggest that sex may contribute to responses to adenosine-targeting agents.

**Abstract:**

The ecto-nucleotidase CD73 is an important immune checkpoint in tumor immunity that cooperates with CD39 to hydrolyze pro-inflammatory extracellular ATP into immunosuppressive adenosine. While the role of CD73 in immune evasion of solid cancers is well established, its role in leukemia remains unclear. To investigate the role of CD73 in the pathogenesis of chronic lymphocytic leukemia (CLL), Eµ-TCL1 transgenic mice that spontaneously develop CLL were crossed with CD73^−/−^ mice. Disease progression in peripheral blood and spleen, and CLL markers were evaluated by flow cytometry and survival was compared to CD73-proficient Eµ-TCL1 transgenic mice. We observed that CD73 deficiency significantly delayed CLL progression and prolonged survival in Eµ-TCL1 transgenic mice, and was associated with increased accumulation of IFN-γ^+^ T cells and effector-memory CD8^+^ T cells. Neutralizing IFN-γ abrogated the survival advantage of CD73-deficient Eµ-TCL1 mice. Intriguingly, the beneficial effects of CD73 deletion were restricted to male mice. In females, CD73 deficiency was uniquely associated with the upregulation of CD39 in normal lymphocytes and sustained high PD-L1 expression on CLL cells. In vitro studies revealed that adenosine signaling via the A2a receptor enhanced PD-L1 expression on Eµ-TCL1-derived CLL cells, and a genomic analysis of human CLL samples found that PD-L1 correlated with adenosine signaling. Our study, thus, identified CD73 as a pro-leukemic immune checkpoint in CLL and uncovered a previously unknown sex bias for the CD73-adenosine pathway.

## 1. Introduction

Chronic lymphocytic leukemia (CLL) is the most common type of leukemia in the Western world [1,2]. CLL mainly affects men and is characterized by the clonal expansion of mature CD5-expressing B cells in blood, spleen, lymph nodes, and bone marrow. While targeted therapies with anti-CD20 monoclonal antibodies (i.e., rituximab, obinutuzumab, ofatumumab, ublituximab) and ibrutinib have substantially ameliorated prognosis, CLL remains mostly unpredictable and is largely incurable when patients become refractory to these treatments. 

CLL is associated with immune dysfunctions, which ultimately promote tumor tolerance and progression. Notably, anti-tumor T cells of CLL patients present an exhausted phenotype [3,4] and targeting the PD-1/PD-L1 axis can promote anti-CLL responses in mice [5]. Accordingly, clinical trials are testing anti-PD-1/PD-L1 immunotherapy in CLL patients [6,7,8].

Adenosine is an immunosuppressive metabolite generated from extracellular ATP, notably by ectonucleotidases CD39 and CD73. CD39 converts ATP into ADP and AMP, while CD73 hydrolyzes AMP into adenosine. Adenosine mainly exerts its immunosuppressive effect through the activation of high-affinity A2a adenosine receptors [9]. Targeting the adenosinergic axis is currently being pursued for the treatment of solid tumors, including in a randomized phase 3 trial for lung cancer (NCT05221840). Despite the established immunosuppressive effects of CD73 in solid tumors, its role in leukemia remains unclear. 

In human CLL, CD73 expression has been associated with increased expression of biomarkers associated with poor prognosis, such as CD38, ZAP-70, and beta-2-microglobulin [10,11]. Human CLL cells also express higher levels of the A2a adenosine receptor than normal B cells, and exogenous adenosine has been shown to protect CLL cells from spontaneous or drug-induced apoptosis [12]. By favoring type 2 macrophage polarization and Tregs accumulation, adenosine may also support the lymphoid niches where CLL cells proliferate [12]. Interestingly, in an adoptively transferred CLL mouse model, A2a blockade was shown to rescue CD8 T cell functions and to prevent Treg expansion [13]. Nevertheless, the specific role of CD73 in the pathogenesis of CLL is unclear. In this study, we investigated the impact of CD73 in the development of CLL in Eµ-TCL1 transgenic mice, a well-validated mouse model of human CLL [14].

## 2. Materials and Methods

### 2.1. Animal Experimentation

Eµ-TCL1 transgenic mice (Eµ-TCL1^tg/wt^ on the C57Bl/6 background), which express the TCL1 oncogene under the IGVH promoter, driving clonal expansion of CD5^int^ B cells [14], were kindly provided by Dr. Carlo Croce (Ohio State University, Columbus, OH, USA) and crossed with CD73^−/−^ C57Bl/6 mice, originally obtained from Dr. Linda Thompson (OMRF, Oklahoma City, OK, USA). Genotyping was performed on ear biopsies harvested upon weaning. Primers are listed in Supplemental Table S1. Birth rate, birth weight, and gender distribution for Eµ-TCL1^tg/wt^ CD73^−/−^ breeding were not different than those of Eµ-TCL1^tg/wt^ mice. For certain experiments, littermates that were revealed to not have integrated the TCL1 transgene upon genotyping were used as non-leukemic, healthy (hWT and hCD73^−/−^) controls. Disease progression was monitored by analysis of peripheral blood composition by FACS and sick mice were sacrificed prior to the apparition of a moribund state. For some experiments, 4- and 8-month-old mice were sacrificed for spleen single-cell analysis. For interferon gamma (IFN-γ) neutralization in vivo, 2-month-old mice were treated intraperitoneally with 200 µg of the anti-IFN-γ monoclonal antibody (clone H22; BioXCell) twice a week for 10 weeks. Animal experimentations were performed in accordance with guidelines from the Canadian Council on Animal Care (CCAC) and were approved by an Institutional Animal Care and Use Committee.

### 2.2. Reagents and Cell Culture

The following reagents were used for the study: 5′-*N*-ethylcarboxamidoadenosine (NECA; Tocris, Bristol, UK, #1691), SCH 58261 (Tocris, #2270), CGS 21680 hydrochloride (Tocris, #1063), recombinant mouse IL-10 protein (R&D systems, #417-ML), and mouse IFN-gamma recombinant protein (Thermofisher, Waltham, MA, USA, #BMS326). CLL cells were isolated from the spleens of deceased Eµ-TCL1^tg/wt^ mice by generating single-cell suspensions. CLL purity was verified by FACS. For in vitro experiments, CLL cells were exposed to either CGS 21680 (1 µM) or NECA (1 µM) with or without SCH 58261 (1 µM) for 48 h at 37 °C in RPMI containing 10% FBS, 100 U/mL penicillin, and 0.1 mg/mL streptomycin, in the presence or absence of recombinant mouse IL-10 protein (100 ng/mL) or recombinant mouse IFN-gamma (10 ng/mL). After 48 h of incubation, PD-L1 expression levels upon treatments were analyzed by FACS (Section 2.4).

### 2.3. Genomic and Transcriptomic Analyses

For analysis of CD73 and CD39 expression in young and healthy C57Bl/6 mice, raw-count RNA sequencing gene expression data of bulk CD19+ thymic B cells (GEO: GSE107110) [15], purified CD4+ naive T cells (CD3+CD4+CD25-CD62L+CD44−), and effector memory (CD3+CD4+CD25-CD62L-CD44+) T cells (GEO: GSE184496) [16] were retrieved from the Gene Expression Omnibus (GEO) portal. Gene expression was computed using the log2-transformed TPM (transcripts per million) method. Differential expression of Nt5e and Entpd1 expression in young vs. old mice were investigated using the DESeq2 R package [17].

For adenosine signaling analysis in human CLL patients, processed gene expression profile (RNA sequencing) and mutation information data (whole-exome sequencing) from a cohort of 156 CLL patients [18,19] were retrieved from the cBioPortal data repository [18,19]. The processed gene expression profile of this cohort was composed of log2-transformed TPM (transcripts per million) data. For further information on data processing, please refer to the original publication from Landau and colleagues [18,19].

### 2.4. Flow Cytometry

Single-cell suspensions were incubated with fluorescence-conjugated antibodies for 30 min at 4 °C and then acquired with a BD LSRFortessa flow cytometer. For cytokine release assay, splenocytes from 8-month-old Eµ-TCL1 WT and CD73^−/−^ mice were cultured with brefeldin A (5 µg/mL; Sigma, St. Louis, MO, USA) alone (unstim) or with PMA (25 ng/mL; Sigma) and ionomycin (1 µg/mL; Sigma) in RPMI (10% FBS, 1% pen/strep, 50 µM 2-mercaptoethanol). After 6 h, media was washed, cells were stained for extracellular markers (CD3, CD4, CD8), fixed, and permeabilized (eBioscience, #00-5523-00), and stained intracellularly. Antibodies used are listed in Supplemental Table S2.

### 2.5. Statistical Analysis

Mann–Whitney and ANOVA tests were performed when comparing 2 and 3 groups, respectively. Šidák corrections were applied to multiple comparisons. Correlations and survival analyses were performed by Pearson and log-rank tests, respectively. All statistical analyses were performed using GraphPad Prism software (version 9.0.2).

## 3. Results

### 3.1. CD73 and CD39 Are Upregulated on Non-Leukemic Lymphocytes upon CLL Progression

**Figure 1:**
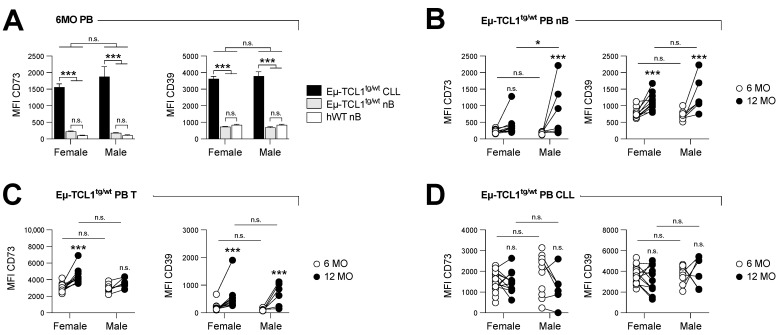
CD73 and CD39 expression is upregulated on non-leukemic lymphocytes upon disease progression. Peripheral blood (PB) cells of male (M) and female (F) Eµ-TCL1^tg/wt^ mice were analyzed at 6 months old (Mo) (early stage; *n* = 25 F and *n* = 11 M) and 12 months old (Mo) (advanced stage; *n* = 11 F and *n* = 6 M). CD73 (left) and CD39 (right) expression levels are shown by mean fluorescence intensity (MFI). (**A**) CD73 and CD39 expression is compared between 6-month-old PB leukemic cells (CLL; CD5^int^ B220^int^) and normal B cells (nB; CD5^neg^ B220^hi^) from Eµ-TCL1^tg/wt^ and hWT mice (*n* = 5 M and 5 F). (**B**–**D**) CD73 and CD39 expression is compared between 6- and 12-month-old (**B**) normal B cells (nB), (**C**) pan-T cells (T; CD5^hi^ B220^neg^) and (**D**) leukemic cells (CLL) of Eµ-TCL1^tg/wt^ mice. Means +/− SEM are shown (* *p* < 0.05; *** *p* < 0.001 by 2-way ANOVA). MO, months; PB, peripheral blood; n.s., non-significant; nB, normal B cells; MFI, mean fluorescence intensity; hWT, healthy WT.

We firstly analyzed CD73 and CD39 protein expression on circulating leukemic cells (CD5^int^ B220^int^), normal B cells (nB cells: CD5^neg^ B220^hi^), and T cells (CD5^hi^ B220^neg^) from Eµ-TCL1^tg/wt^ mice (Figure S1A–D). Both CD73 and CD39 were found to be significantly upregulated on leukemic CLL cells compared to nB (Figure 1A) in the early stage of the disease (i.e., 6-month-old mice). The expression levels of CD73 and CD39 were further increased upon disease progression on nB cells and T cells (Figure 1B,C), while leukemic CLL cells maintained high levels of both ectonucletotidases (Figure 1D). Similar expression levels were observed in the spleens (Figure S1E–H). By contrast, lymphocytes from aged, healthy control mice did not upregulate CD73 and CD39 to the same extent as CLL-bearing mice (Figure S1I–K).

### 3.2. CD73 Deficiency Prolongs Survival of Eµ-TCL1 Male Mice in an IFN-γ-Dependent Manner

**Figure 2:**
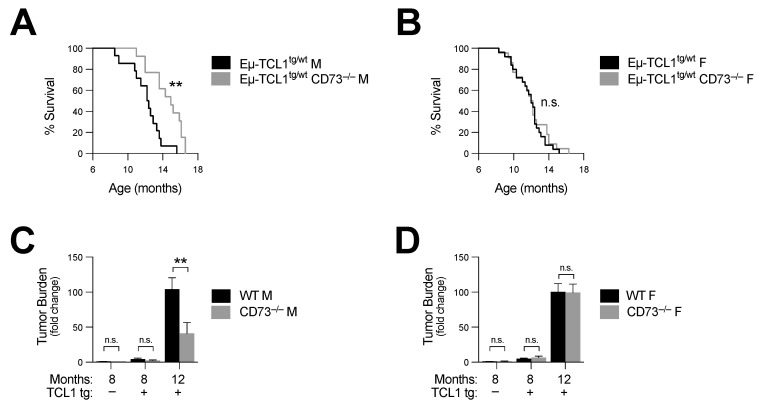
CD73 deficiency prolongs survival of Eµ-TCL1^tg/wt^ males. Eµ-TCL1^tg/wt^ mice were crossed with CD73^−/−^ mice and leukemia progression was analyzed. (**A**,**B**) Survival of Eµ-TCL1^tg/wt^ (*n* = 14 M and 25 F) and Eµ-TCL1^tg/wt^ CD73^−^^/−^ (*n* = 13 M and 22 F) (**a**) males and (**B**) females. (**C**,**D**) Fold change in peripheral disease burden of Eµ-TCL1^tg/wt^ and Eµ-TCL1^tg/wt^ CD73^−/−^ (**C**) 8- and 12-month-old male and (**D**) female mice relative to 8-month-old hWT mice (hWT *n* = 5 M and 5 F; hCD73^−/−^ *n* = 7 M and 5 F). Means +/− SEM are shown (** *p* < 0.01 by log-rank (**A**,**B**) or Mann–Whitney test (**C**,**D**)). F, female; M, male; tg, transgenic; n.s., non-significant.

Next, we crossed Eµ-TCL1^tg/wt^ mice with CD73^−/−^ mice and analyzed disease progression and survival. We observed that CD73 deficiency significantly increased the survival of male CD73^−/−^ Eµ-TCL1 mice (log-rank *p* = 0.002) (Figure 2A and Table 1). Consistent with this observation, peripheral disease burden was also significantly reduced at 12 months (Figure 2C and Figure S2A). Surprisingly, no survival difference was observed in female mice (log-rank *p* = 0.54) (Figure 2B and Table 1). CD73-deficient female Eµ-TCL1^tg/wt^ mice showed a similar disease burden to that of control female mice (Figure 2D and Figure S2B).

**Figure 3:**
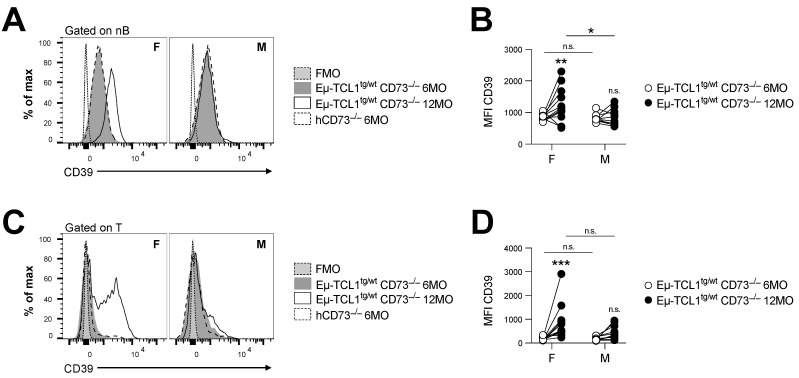
CD73-deficient Eµ-TCL1^tg/wt^ males fail to upregulate CD39 in peripheral blood. Peripheral blood (PB) cells of male (M) and female (F) CD73^−/−^ Eµ-TCL1^tg/wt^ mice were analyzed at 6 (*n* = 22 F and *n* = 13 M) and 12 (*n* = 12 F and *n* = 10 M) months old (MO). CD39 expression levels shown by mean fluorescence intensity (MFI) on PB (**A**,**B**), normal B cells (nB; CD5^neg^ B220^hi^) and (**C**,**D**) pan-T cells (T; CD5^hi^ B220^neg^). Six-month-old hCD73^−/−^ littermates were used as control. Means +/− SEM are shown (* *p* < 0.05; ** *p* < 0.01; *** *p* < 0.001 by 2-way ANOVA). FMO, fluorescence minus one; MO, months; hCD73^−/−^, healthy CD73^−/−^; nB, normal B cells; n.s., non-significant; MFI, mean fluorescence intensity.

CLL is known to alter T cell function to promote immune escape [20,21]. We, thus, evaluated whether loss of CD73 in female Eµ-TCL1^tg/wt^ mice was associated with a gain in potential compensatory pathways. Firstly, we observed that CD73 deficiency in female Eµ-TCL1^tg/wt^ mice was associated with significant upregulation of CD39 expression in normal lymphocytes (Figure 3). This was not observed in male mice, who did not reflect a change in the frequency of CD4^+^ Foxp3^+^ T regulatory cells (Figure S2C), known to express CD39.

**Figure 4:**
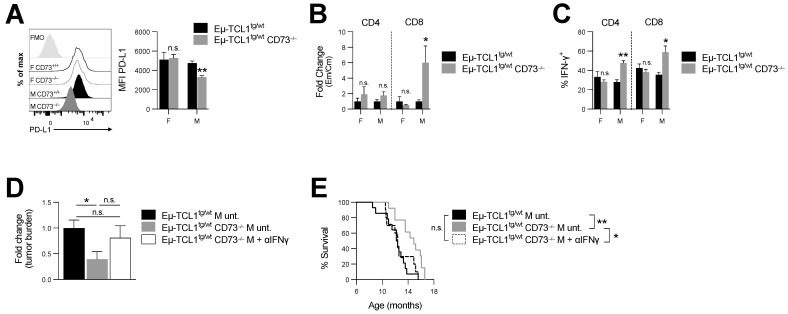
CD73^−/−^ Eµ-TCL1^tg/wt^ males display increased antitumor immunity and IFN-γ neutralization abrogates their prolonged survival. Spleens’ compositions of 8-month-old Eµ-TCL1^tg/wt^ and Eµ-TCL1^tg/wt^ CD73^−/−^ male and female mice were analyzed by cytometry. (**A**) Analysis of PD-L1 expression levels (MFI) on splenic leukemic CLL (CD5^int^ B220^int^) cells (M Eµ-TCL1^tg/wt^ *n* = 7; M Eµ-TCL1^tg/wt^ CD73^−/−^ *n* = 8; F Eµ-TCL1^tg/wt^ *n* = 8; F Eµ-TCL1^tg/wt^ CD73^−/−^ *n* = 7). (**B**) Fold change in ratios of effector memory (Em: CD44^+^CD62L^-^) to central memory (Cm: CD44^+^CD62L^+^) CD4+ and CD8+ (gated on live CD3+) splenic T cells in Eµ-TCL1^tg/wt^ and Eµ-TCL1^tg/wt^ CD73^−/−^ (M Eµ-TCL1^tg/wt^ *n* = 17; M Eµ-TCL1^tg/wt^ CD73^−/−^ *n* = 19; F Eµ-TCL1^tg/wt^ *n* = 9; F Eµ-TCL1^tg/wt^ CD73^−/−^ *n* = 10). (**C**) Percentages of IFN-γ^+^ CD4 and CD8 T cells from 8-month-old Eµ-TCL1^tg/wt^ and Eµ-TCL1^tg/wt^ CD73^−/−^ splenocytes stimulated in vitro with PMA/ionomycin for 6h (M Eµ-TCL1^tg/wt^ *n* = 5; M Eµ-TCL1^tg/wt^ CD73^−/−^ *n* = 5; F Eµ-TCL1^tg/wt^ *n* = 6; F Eµ-TCL1^tg/wt^ CD73^−/−^ *n* = 6). (**D**) Fold change in peripheral disease burden relative to untreated Eµ-TCL1^tg/wt^ mice and (**E**) survival of Eµ-TCL1^tg/wt^ CD73^−/−^ males treated with anti-interferon gamma (αIFNγ; *n* = 10) compared to historical untreated (unt.) Eµ-TCL1^tg/wt^ (*n* = 14) and Eµ-TCL1^tg/wt^ CD73^−/−^ (*n* = 13) controls. Means +/− SEM are shown (* *p* < 0.05; ** *p* < 0.01 by Mann–Whitney test (**A**–**C**), 1-way ANOVA (**D**) and log-rank (**E**)). FMO, fluorescence minus one; F, female; M, male; Em, effector memory; Cm, central memory; unt., untreated; n.s., non-significant.

Secondly, since CLL cells can express high levels of PD-L1, and PD-L1 blockade can restore antitumor immunity in Eµ-TCL1 mice [5,21], we also compared PD-L1 expression levels on leukemic cells in male versus female CD73-deficient Eµ-TCL1^tg/wt^ mice. We observed that, in contrast to male mice, which downregulated PD-L1 expression on CLL cells (Figure 4A), CD73-deficient female mice maintained high levels of PD-L1 on CLL and normal B cells (Figure 4A and Figure S2D).

Our data thus suggest that, while CD73 deficiency was associated with greater antitumor immune surveillance in male Eµ-TCL1^tg/wt^ mice, female mice upregulated immunosuppressive CD39 and PD-L1 upon CD73 deletion. Further, in support of an important role for CD73 in regulating anti-CLL tumor immunity, and consistent with the fact that CD8^+^ effector memory T cells (T_EM_) have been shown to restrain CLL progression [22], loss of CD73 was also associated with a significant increase in CD8^+^ T_EM_/T_CM_ (Figure 4B and Figure S2E,F) and in IFN-γ production in male Eµ-TCL1^tg/wt^ mice (Figure 4C and Figure S2G).

Because IFN-γ is an important component of the anti-leukemic response [23], next, we evaluated whether IFN-γ was required for the anti-CLL activity associated with CD73 targeting. For this purpose, Eµ-TCL1^tg/wt^ CD73^−/−^ mice were treated with anti-IFN-γ neutralizing mAb and disease progression was monitored over time. CD73-deficient Eµ-TCL1^tg/wt^ male mice, injected with a neutralizing anti-IFN-γ mAb, were no longer protected from CLL (Figure 4D,E and Figure S2H and Table 1). Accordingly, neutralizing IFN-γ increased tumor burden (albeit not significantly; Figure 4D) and significantly decreased survival of CD73-deficient Eµ-TCL1^tg/wt^ male mice (Figure 4E).

### 3.3. A2a Adenosine Receptor Signaling Drives PD-L1 Expression on CLL Cells

**Figure 5:**
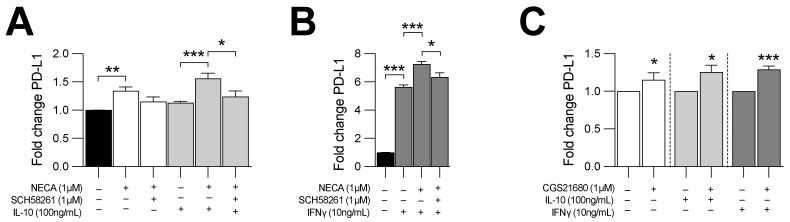
A2a adenosine receptor signaling potentiates PD-L1 expression on Eµ-TCL1-derived CLL cells. CD73-deficient Eµ-TCL1-derived CLL cells were cultured in vitro for 48 h and PD-L1 expression was analyzed by FACS. (**A**,**B**) MFI and fold change in PD-L1 expression of CD73-deficient Eµ-TCL1 (male)-derived CLL cells upon exposition to NECA (1µM; *n* = 3) +/− SCH58261 (1µM; *n* = 2) in presence or not of (**A**) mouse recombinant IL-10 (100 ng/mL) or (**B**) mouse recombinant IFN-γ (10 ng/mL). (**C**) MFI and fold change in PD-L1 expression of CLL cells derived from 3 Eµ-TCL1^tg/wt^ CD73^−/−^ male mice exposed to CGS 21680 (1 µM; *n* = 2). Means +/− SEM are shown (* *p* < 0.05; ** *p* < 0.01; *** *p* < 0.001 by 1-way ANOVA).

We investigated whether adenosine signaling regulated PD-L1 expression on leukemic cells. CLL cells were treated with NECA, a stable adenosine receptor agonist, with or without exogenous IL-10 and IFN-γ, which have been shown to modulate PD-L1 expression on CLL [24,25]. Remarkably, treatment with NECA significantly increased PD-L1 expression on CLL cells, an effect further exacerbated when cells were co-treated with IL-10 or IFN-γ (Figure 5A,B). We hypothesized that the effect of NECA was due to the activation of A2a adenosine receptors. Accordingly, NECA-mediated upregulation of PD-L1 on CLL cells was abrogated upon addition of the selective A2a receptor antagonist SCH 58261, (Figure 5A,B). Consistent with this, treatment of CLL cells with the A2a receptor agonist CGS 21680 significantly upregulated PD-L1 expression (Figure 5C). Taken together, our results indicate that adenosine signaling via A2a receptors contributes to high PD-L1 expression levels on CLL.

### 3.4. Adenosine Signaling Is Associated with Increased PD-L1 Expression

**Figure 6:**
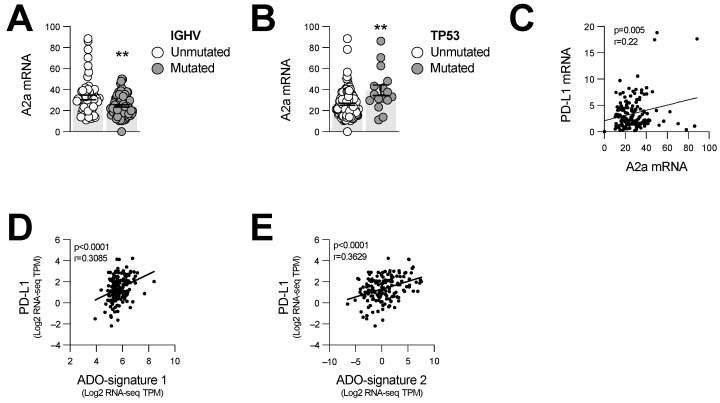
ADORA2A gene expression is associated with PD-L1 levels and increased risk stratification. Gene expression profile and mutation information data from 156 CLL patients, publicly available through the cBio Cancer Genomics Portal, were used to compared mRNA levels (RNA-seq TPM) of A2a (ADORA2A) between high- and low-risk patients. (**A**) A2a gene expression levels in IGHV-mutated (low risk; *n* = 92) and -unmutated (high risk; *n* = 59) patients. (**B**) A2a gene expression levels in TP53-mutated (high risk; *n* = 16) and -unmutated (low risk; *n* = 140) patients. (**C**) Correlation between A2a and PD-L1 gene expression. (**D**,**E**) Correlations between PD-L1 mRNA and adenosine gene signatures published by (**D**) Sidders et al. [26] (ADO-signature 1) and (**E**) Fong et al. [27] (ADO-signature 2). Means +/− SEM are shown (** *p* < 0.01; by Mann–Whitney test (**A**,**B**) and Pearson correlation (**C**–**E**)). TPM, transcript per million.

We finally evaluated whether the expression levels of A2a adenosine receptors were associated with high-risk IGHV-unmutated or TP53-mutated CLL. For this, we analyzed 156 human CLL samples with available gene expression profile and mutation information data [18,19]. In support of our mouse data, higher A2a (ADORA2A) gene expression was significantly associated with IGHV-unmutated and TP53-mutated CLL (Figure 6A,B). Moreover, ADORA2A gene expression levels positively correlated with increased PD-L1 (CD274) gene expression (Figure 6C). Two previously reported adenosine-associated gene expression signatures [26,27] were also positively correlated with PD-L1 levels in CLL cells (Figure 6D,E). Our results thus suggest an important role for the CD73-adenosine-A2a axis in promoting immune escape of CLL.

## 4. Discussion

CLL is characterized by a dysfunctional anti-tumor immune response [3,5,21]. Preclinical studies suggest that immunotherapy with an immune checkpoint blockade can promote anti-CLL immunity [5,13]. Using the Eµ-TCL1 transgenic mouse model, we have demonstrated an important role for the CD73-adenosine axis in CLL. Our findings are in agreement with a previous study on A2a-mediated immune dysfunction in CLL [13], and further support that targeting the CD73-adenosine immune checkpoint may be a therapeutic avenue in CLL.

An important finding of our study is that male Eµ-TCL1^tg/wt^ CD73^−/−^ mice exhibited enhanced immune surveillance against CLL, which was associated with reduced PD-L1 expression on CLL cells. Mechanistically, this was associated with increased generation of effector memory CD8+ T cells and increased IFN-γ production. Adenosine produced by the CD73-activated A2a adenosine receptor on CLL cells enhanced PD-L1 expression. The A2a adenosine receptor is highly expressed on CLL cells [10]. Notably, cAMP signaling has been shown to enhance PD-L1 expression on diffuse large B cells’ leukemia (DLBCL) cells [24]. Our data thus suggest that A2a receptor-mediated cAMP accumulation also promotes PD-L1 expression on CLL cells. In support of this, we observed a strong positive correlation between PD-L1 expression and A2a receptors or adenosine-regulated genes in human CLL [26,27]. 

The underlying mechanisms explaining the observed sex bias remain unclear. Intriguingly, a recent study reported female-specific upregulation of CD39 in hepatocytes upon CD73 gene deletion [28]. Upregulation of CD39 may favor alternative adenosine-generating pathways. It remains unclear whether adenosine signaling is regulated by sex-specific hormones. Further studies should dissect the impact of gonadectomy on the expression of adenosine signaling regulatory proteins (e.g., CD39, CD73, and A2a receptors).

Our observation of female-specific increases in CD39 expression on B cells may reflect a sex-specific accumulation of IL-10-secreting Bregs, as was documented in humans [29]. While the impact of adenosine signaling on IL-10 production during CLL remains unknown, cAMP accumulation was recently described to favor IL-10 production in DLBCL [24]. IL-10 notably plays a critical role in CLL pathogenesis [30,31] and in the regulation of PD-L1 expression [24,25]. Sex-mediated differences in IL-10 production, as described in solid tumors [32], may thus contribute to the sex bias of CD73-deficient Eµ-TCL1^tg/wt^ mice.

It should be emphasized that the observed increase in median survival of CD73-deficient versus CD73-proficient Eµ-TCL1^tg/wt^ male mice (i.e., nearly 3 months) is in the same range as what has been reported for ibrutinib treatment in this model [33]. CD73 deficiency did not simply exacerbate a preexisting survival sex bias in the Eµ-TCL transgenic mouse model, as reported by Koch et al. [34]. We did not observe such bias in our studies. 

Finally, our study comprises important limitations, notably regarding the translation of results obtained from mouse studies to human patients with CLL. CD73 biology may differ between mice and humans as CD73^−/−^ mice do not fully recapitulate CD73 deficiency in humans [35,36]. In addition, since disease severity is greater in men with CLL [2], but slightly milder in male Eµ-TCL1 transgenic mice [34], aspects of CLL pathogenesis may not be fully recapitulated by the mouse model used in this report. 

## 5. Conclusions

Overall, our study identifies an important role for the CD73-adenosine axis in promoting CLL progression and immune escape, notably by increasing PD-L1 expression on CLL cells. While the use of the Eµ-TCL1 transgenic mouse model to study the role of CD73 in CLL pathogenesis may not fully recapitulate human disease, our findings prompt further investigation of the therapeutic potential of targeting CD73 in CLL, especially in men.

## Figures and Tables

**Table 1:** Median survival time of experimental groups of Eµ-TCL1^tg/wt^ transgenic mice.

Experimental Group	Sex	*n*	Survival (Months)	
			Median	Range
Eµ-TCL1^tg/wt^	F	25	12.0	8.3–15.2
	M	14	12.3	8.5–15.6
Eµ-TCL1^tg/wt^ CD73^−/−^	F	22	12.1	8.3–16.3
	M	13	14.9	11.0–16.6
Eµ-TCL1^tg/wt^ CD73^−/−^ + anti-IFN-γ	M	10	12.4	10.5–15.6

## Data Availability

The data presented in this study are available on request from the corresponding author.

## Supplementary Materials

The following supporting information can be downloaded at: https://www.mdpi.com/article/10.3390/cancers14133130/s1, Figure S1: Characterization of CLL disease progression and CD73 and CD39 expression in Eμ-TCL1 transgenic mice; Figure S2: Comparison of CLL disease progression between Eμ-TCL1^tg/wt^ and Eμ-TCL1tg/wt CD73^−/−^ mice; Table S1: Primers used for genotyping Eµ-TCL1 tg and CD73^−/−^ breedings; Table S2: Fluorescence-conjugated antibodies used for FACS analyses.
