# Supplementary material for: CD73 Promotes Chronic Lymphocytic Leukemia
Source: Cancers (Basel). 2022 Jun 26;14(13):3130. doi: 10.3390/cancers14133130 (PMC9264813; doi:10.3390/cancers14133130)
Supplement: Supplementary file 1 [file cancers-14-03130-s001.zip › Supplementary-Figures S1 and S2.pdf]

Supp. Figure S1

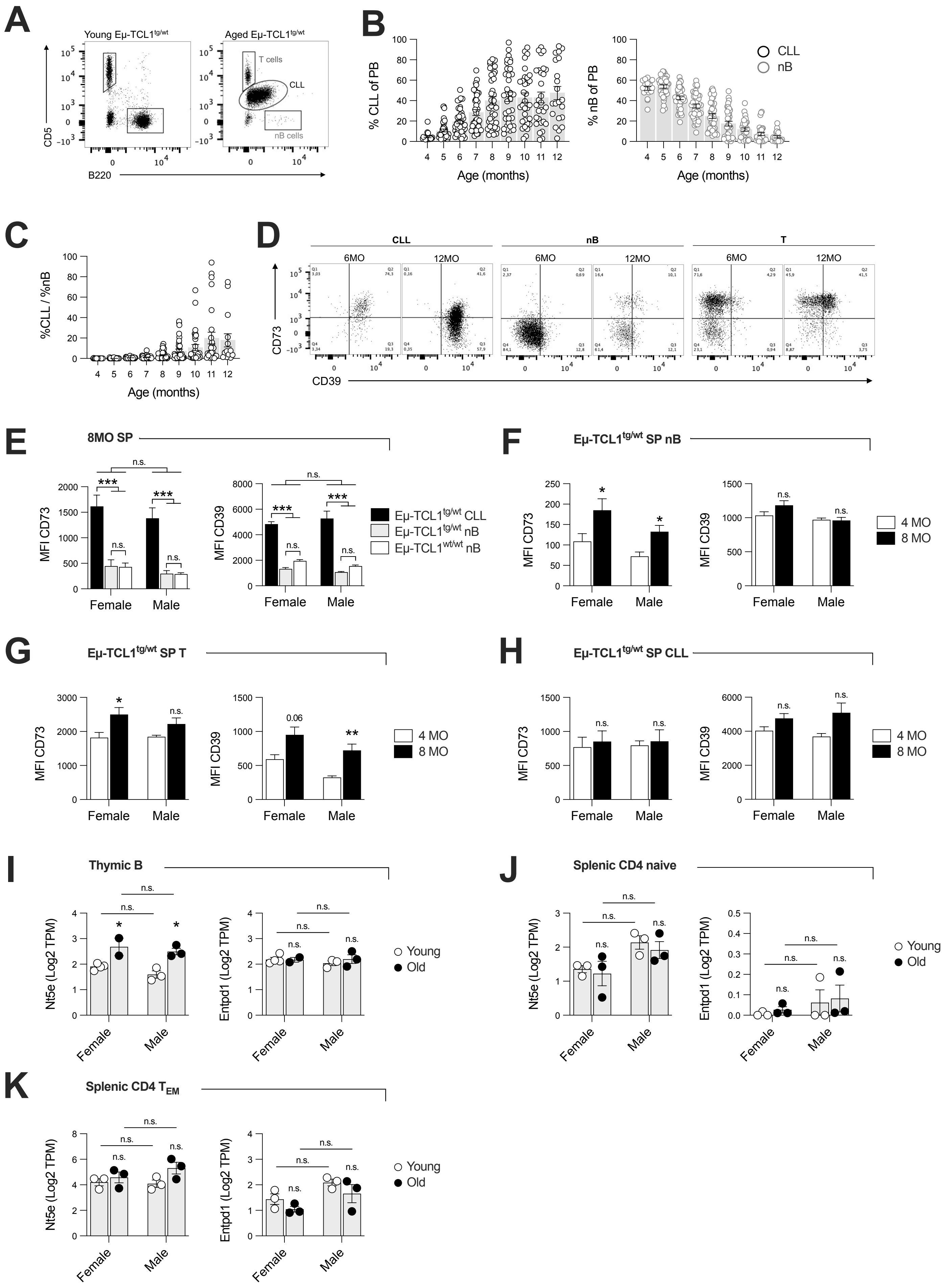

Figure S1. Characterization of CLL disease progression and CD73 and CD39 expression in Eμ-TCL1 transgenic mice. A Peripheral blood (PB) from young (2 months-old) and aged (12 months-old) Eμ-$TCL1^{tg/wt}$ mice was analyzed by flow cytometry using anti- CD5 and B220 fluorescent antibodies. Example of the gating strategy employed to delineate circulating populations is shown. **B** Percentage of leukemic cells (CLL) and normal B cells (nB) in PB of Eμ-$TCL1^{tg/wt}$ mice over time. **C** Ratio of leukemic CLL cells over nB cells populations in PB of Eμ-$TCL1^{tg/wt}$ mice over time. N=14 males and 22 females. **D** Representative raw FACS plot for CD73 and CD39 expression from paired Eμ-$TCL1^{tg/wt}$ 6 and 12 months-old peripheral leukemic cells (CLL; left panels), normal B cells (nB; middle panels) and T cells (T; right panels). **E-H** Spleens (SP) of male (M) and female (F) Eμ-$TCL1^{tg/wt}$ mice was analyzed at 4- (early stage; n=5 F and n=5 M) and 8- (advanced stage; n=5 F and n=5 M) months-old (MO). CD73 (left) and CD39 (right) expression levels are showed by mean fluorescence intensity (MFI). **E** CD73 and CD39 expression is compared between 4 months-old splenic leukemic CLL cells and nB cells from Eμ-$TCL1^{tg/wt}$ mice and nB cells from age-matched Eμ-$TCL1^{wt/wt}$ littermate controls. **F-H** CD73 and CD39 expression is compared between 4 and 8 months-old (**F**) nB cells, (**G**) pan-T cells (T) and (**H**) leukemic CLL cells. **I-K** CD73 (*Nt5e*) and CD39 (*Entdp1*) gene expression levels were compared between young and old healthy C57Bl/6 mice. For the thymic B cell cohort (**I**)**,** mice were classified as young (age ≤ 8 weeks) and old (age ≥ 40 weeks). For the CD4+ T cell cohort (naive CD4 T cells (**J**) and effector memory T cells (**K**)), mice were classified as young (age ≤ 11 weeks) and old (age ≥ 83 weeks). Means +/- SEM are shown (*$p<0.05$; **$p<0.01$; ***$p<0.001$ by 2-way ANOVA). nB, normal B cells; PB, peripheral blood; MO, months; SP, spleen; MFI, mean fluorescence intensity; TPM, transcripts per million; n.s., non-significant.

Supp. Figure S2

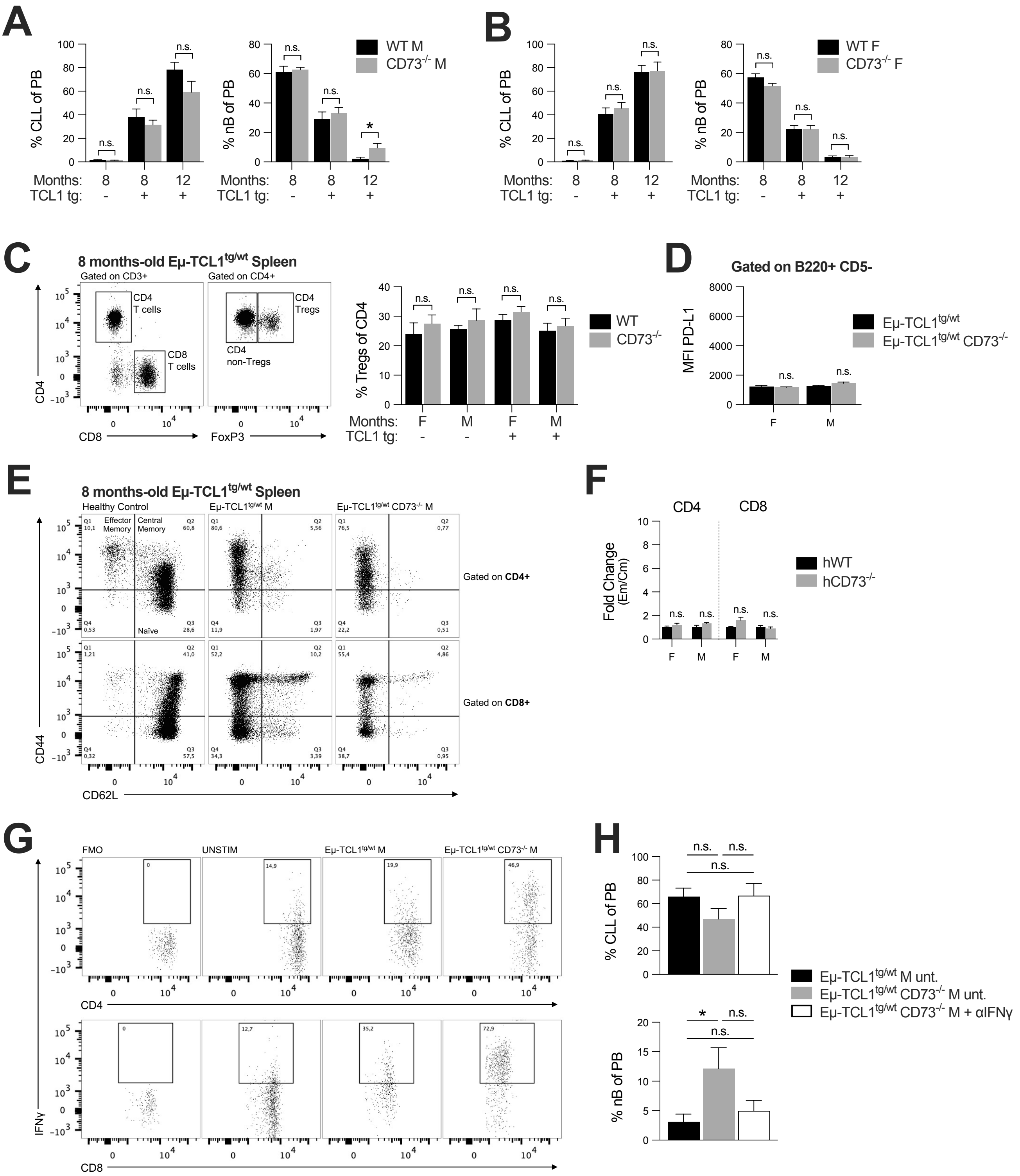

Figure S2. Comparison of CLL disease progression between Eμ-TCL1$^{tg/wt}$ and Eμ-TCL1$^{tg/wt}$ CD73$^{-/-}$ mice. Eμ-TCL1$^{tg/wt}$ mice were crossed with CD73$^{-/-}$ mice and leukemia progression was analyzed. **A-B** Percentages of circulating leukemic cells (CLL; left panels) and normal B cells (nB; right panels) in peripheral blood (PB) of Eμ-TCL1$^{tg/wt}$ and Eμ-TCL1$^{tg/wt}$ CD73$^{-/-}$ males (**A**) and females (**B**) aged 8 and 12 months-old (complement to figure 2). Non-leukemic hWT and hCD73$^{-/-}$ mice are shown as healthy controls. **C-F** 8 months-old Eμ-TCL1$^{tg/wt}$ and Eμ-TCL1$^{tg/wt}$ CD73$^{-/-}$ male (M) and female (F) mice were sacrificed, and spleens' single cell suspensions were analyzed by cytometry (complement to figure 4A-B). Non-leukemic hWT and hCD73$^{-/-}$ mice are shown as healthy controls. **C** Gating strategy and percentages of FoxP3^{+} Tregs out of CD4 T cells. **D** PD-L1 expression levels (MFI) on normal B cells (nB) of Eμ-TCL1$^{tg/wt}$ mice. **E** Gating strategy to define effector memory (CD44^{+}CD62L^{-}), central memory (CD44^{+}CD62L^{+}) or naïve (CD44^{-}CD62L^{+}) phenotype of CD4 (top panels) and CD8 (bottom panels) splenic T cells. 8 weeks-old C57Bl/6 non-transgenic mice (Jackson laboratories) were used as healthy control. **F** Fold change of ratios of effector-memory (Em: CD44+CD62L-) to central-memory (Cm: CD44+CD62L+) CD4+ and CD8+ (gated on live CD3+) splenic T cells in hWT and hCD73$^{-/-}$ control mice. **G** Representative gating showing percentages of IFNγ^{+} CD4 (top) and CD8 (bottom) T cells upon in vitro stimulation (PMA/iono) for 6 hours (complement to figure 4C). **H** Percentage of circulating leukemic CLL (top) and nB (bottom) cells in anti-IFN gamma-treated CD73$^{-/-}$ Eμ-TCL1$^{tg/wt}$ male mice compared to control untreated Eμ-TCL1$^{tg/wt}$ mice (complement to figure 4D-E). Means +/- SEM are shown (*$p<0.05$; **$p<0.01$ by Mann-Whitney test (**A-D**) and by 1-way ANOVA (**H**)). PB, peripheral blood; M, male; F, female; tg, transgenic; hWT, healthy WT; Em, effector memory; Cm, central memory; FMO, fluorescence minus one; nB, normal B cells; unt., untreated; n.s., non-significant.
